# Data protection, data management, and data sharing: Stakeholder perspectives on the protection of personal health information in South Africa

**DOI:** 10.1371/journal.pone.0260341

**Published:** 2021-12-20

**Authors:** Ciara Staunton, Kathrina Tschigg, Gayle Sherman

**Affiliations:** 1 School of Law, Middlesex University, London, United Kingdom; 2 Institute for Biomedicine, Eurac Research, Bolzano, Italy; 3 Faculty of Medicine and Health Sciences, University of Cape Town, Cape Town, South Africa; 4 Center for HIV and STI, National Institute for Communicable Diseases, a division of the National Health Laboratory Service, Johannesburg, South Africa; 5 Department of Paediatrics and Child Health, Faculty of Health Sciences, University of the Witwatersrand, Johannesburg, South Africa; Universidad Internacional de La Rioja, SPAIN

## Abstract

The Protection of Personal Information Act (POPIA) 2013 came into force in South Africa on 1 July 2020. It seeks to strengthen the processing of personal information, including health information. While POPIA is to be welcomed, there are concerns about the impact it will have on the processing of health information. To ensure that the National Health Laboratory Service [NHLS] is compliant with these new strict processing requirements and that compliance does not negatively impact upon its current screening, treatment, surveillance and research mandate, it was decided to consider the development of a NHLS POPIA Code of Conduct for Personal Health. As part of the process of developing such a Code and better understand the challenges faced in the processing of personal health information in South Africa, 19 semi-structured interviews with stakeholders were conducted between June and September 2020. Overall, respondents welcomed the introduction of POPIA. However, they felt that there are tensions between the strengthening of data protection and the use of personal information for individual patient care, treatment programmes, and research. Respondents reported a need to rethink the management of personal health information in South Africa and identified 5 issues needing to be addressed at a national and an institutional level: an understanding of the importance of personal information; an understanding of POPIA and data protection; improve data quality; improve transparency in data use; and improve accountability in data use. The application of POPIA to the processing of personal health information is challenging, complex, and likely costly. However, personal health information must be appropriately managed to ensure the privacy of the data subject is protected, but equally that it is used as a resource in the individual’s and wider public interest.

## Introduction

Vast quantities of healthcare data are being collected and processed to predict outbreaks, respond to epidemics, improve service delivery in health care systems, and cut costs. Medical research is witnessing exponential growth in the generation of data and analysing these data can gain new insights into health and diseases. To harness the value of these data, there has been a push towards more “open science” and data sharing [1]. The benefits of data sharing in research include the optimal use of a valuable resource, more reproducible science, promotion of new research on existing data sets, and fostering innovation [2]. In the clinical context, it is hoped that genomics can be integrated into public health which relies on access to data, good clinical practice requires the sharing of data, and access to data is important to assess programmatic interventions [3, 4]. In parallel with this push towards data sharing, there has been a strengthening of data protection regulations in many countries across the world, that seeks to promote the free movement of data while protecting the rights of data subjects. The challenge is how to govern the use of health data (i.e. data that relates to the health of an individual) so that it is made available for individual patient care, national health treatment programmes, and for research, but in a manner that safeguards the privacy of data subjects [5].

The South African National Health Laboratory Service (NHLS) is the provider of diagnostic pathology services for the public sector in South Africa and supports the national and provincial departments in the delivery of health care. The National Health Laboratory Service Act No 37 of 2000 mandates the NHLS to provide cost-effective health laboratory services, support health research, and provide training for health science education. In fulfilling this mandate, the NHLS plays a major role in screening, surveillance, treatment, and research. The fulfilment of its duties requires the NHLS to gather data from patients that is collected by NHLS and non-NHLS employed staff, capture data onto a laboratory information system, store data physically and electronically, link data with other data sets, and share data both within and outside of the NHLS. Fulfilment of these duties requires the NHLS to share data with healthcare professions employed both in the public and private sector, university staff, as well international organisations that are involved in the funding and delivery of national treatment programmes such as President’s Emergency Plan For AIDS Relief (PEPFAR). It also requires the NHLS to link their datasets with datasets held by institutions outside of the NHLS.

The South African National Institute for Communicable Diseases (NICD) is a subsidiary of the NHLS that has a particularly important role in HIV surveillance, outbreak response, and treatment. As part of this work, ‘Results for Action (RfA) Reports’ are clinical reports developed to identify patient test results requiring clinical action. Patient laboratory test results and patient identifiers such as name, surname, facility folder number, and date of birth of all patients who have been tested in that time period are collated into one report identifying patients that need to be followed-up. Thus the users of the reports will not only receive the personal information of their patients, but of all patients who have been tested that week. For example, the HIV Polymerase Chain Reaction’ (PCR) RfA report collates diagnostic test results processed in the previous week for a health facility or district to facilitate rapid follow up and linkage to care of newly diagnosed HIV-infected children. Current South African HIV Guidelines recommend various RfA reports as best practice for enabling patient follow-up [6].

The Protection of Personal Information Act (POPIA) No 4 of 2013 came into force in South Africa on 1 July 2020, with a 1 year period of grace granted for compliance. It was introduced to give effect to the right to informational privacy, derived from the constitutional right to privacy [7]. Similar to the EU General Data Protection Regulation (GDPR) and other data protection regulations and legislations introduced in many other African countries, POPIA seeks to regulate the processing of personal information i.e information that relates to an identifiable living person. It sets out 8 conditions that must be met in the processing of personal information in South Africa: accountability, processing limitation, purpose specification, further processing limitation, information quality, openness, security safeguards, and data subject participation. A breach of POPIA may result in a fine, imprisonment or both. Similar to the GDPR, any individual or organisation seeking to process personal information (that includes collecting, storing, sharing or transfer out of South Africa) that is in South Africa must be compliant with POPIA. POPIA is a general legal framework that seeks to regulate the processing of all types of personal information and this includes health information. POPIA provides rules at a high level and not a sector-specific level. While the Office of the Information Regulator (the independent regulator set up under the Act to monitor and enforce compliance) has indicated that guidance in the use of health data in the clinical context may be forthcoming, tailored guidance on the processing of health information is currently lacking.

POPIA is just one of the many legislative frameworks that impacts the processing of personal health information within the NHLS. The National Health Laboratory Service Act No 37 of 2000, the National Health Act No 61 of 2003 and 2012 regulations, the Promotion of Access to Information Act No 2 of 2000, as well as the common law duty of confidentiality, all impact and regulate the processing of personal information held by the NHLS. Outside of this legislative backdrop, data practices and attitudes towards the collection, use and sharing of data within South Africa have also been informed by exploitative research practices, South Africa’s colonial and apartheid past, and more recent examples of misuse of South African research data [8–11]. However, issues around collection, access, sharing, and use of personal health information in South Africa have come to a head with POPIA.

POPIA prohibits the processing of the personal information of children and “special personal information”. Special personal information includes personal information on race, health, sex life, and biometric information. The processing of such information is generally prohibited, unless it falls within one of the authorised purposes. The authorisations include consent of the data subject, processing for research purposes, or for the care and treatment of the data subject. This authorisation is in addition to the 8 conditions that must be met for the processing of personal information under POPIA. Therefore POPIA will impact the way in which the NHLS collects, stores, uses, and shares personal information.

In 2019, with the coming into force of POPIA anticipated, concerns were expressed about the impact of POPIA on the RfA Reports and initially focused on the security of the reports. When the RfA Reports were first introduced, they were circulated by email to all registered healthcare personnel, with users accepting the terms and conditions to maintain confidentiality, in a zipped, unencrypted but generic, password-protected file. Email distribution was replaced with a secure online portal. Approved users were allocated their own unique user name and password (incorporating the capability of a 2 factor authentication process for resetting their passwords) to access the RfA Reports. This served to improve the security of the NICD i*ssuing* these reports. Concerns persisted on the *sharing* of the reports as registered users still need to share the reports with other healthcare and community care workers to enable them to act on the results. This may require registered users printing out and sharing paper copies of the reports, or sharing them via a mobile application as the healthcare or community care worker may not have access to reliable internet and/or computer equipment. The success of the RfA Reports, and by implication, individual patient care and the national HIV treatment program in South Africa, is dependent on this onward sharing of the reports.

There were concerns that the healthcare workers and the NICD may continue to be at risk of non-compliance with POPIA and a legal analysis of the system was conducted in 2019 to determine whether the new online portal would be compliant with POPIA once in force. This review found that while there was considerable improvement in this new system, the RfA Report system was still not compliant with POPIA. In particular it found that the system was in breach of the following POPIA conditions: processing limitation, purpose specification, information quality, and security safeguards. The review issued a number of recommendations including applying to the Information Regulator to exempt the RfA Reports from some of the conditions for the processing of personal information (as per section 37 of POPIA) and updating its standard operating procedures (SOP) to educate users on using the RfA Reports in a manner that is compliant with POPIA. Outside of the RfA Reports, the review recommended the development of a POPIA Code of Conduct for the use of personal information by the NHLS.

Chapter 7 of POPIA permits the development of codes of conduct for specified personal information or a class of personal information such as health information, a specific activity such as research, or a specific industry, profession or vocation. The development of similar codes of conduct are underway where similar data protection regulations are in force. Notably in the EU, codes of conduct for health research, for service providers in clinical research, and cloud security (amongst others) are currently being drafted. For the NHLS, the development of a Code would provide detailed guidance to the NHLS, its staff, and all external individuals and organisations (including international organisations) that require access to NHLS data on complying with POPIA.

Arising from this review, it was decided to train NICD and NHLS staff on POPIA and provide ongoing information lectures; to apply for exemptions to certain conditions in POPIA for the RfA Reports; to apply for prior authorisation to the Information Regulator so that NHLS datasets could be linked with datasets held by bodies outside of the NHLS; and to develop a Code of Conduct for the NHLS. It is the development of the Code of Conduct for the NHLS (hereinafter referred to as “the Code”) that is the subject of this paper.

In February 2020 the Information Regulator issued guidelines on the development of a Code mandating that all relevant stakeholders be afforded the opportunity to comment on any Code prior to its submission for approval to the Information Regulator. Previous work on stakeholder engagement in South Africa made it clear that there are three layers of engagement that are important: high-level engagement (that includes funders, policymakers, and government officials); peer engagement (that includes healthcare professionals, scientists, and researchers); and community engagement (that includes patients and research subjects) [12]. With this in mind, we sought to first engage with high level and peer-level stakeholders in the assessment of requirements for the code.

At the time of the interviews no other codes on the use of health information were in development and thus the interviews discussed the use of personal information in the context of clinical care, treatment programmes, and research. Since then, the Academy of Sciences of South Africa (ASSAf) has embarked on a process to develop a POPIA Code of Conduct of Research that (once approved by the Information Regulator) will provide guidance on the use of personal information for research in South Africa [13]. These interviews also occurred prior to the gazetting of the draft National Data and Cloud Policy (to be discussed below) but many of the issues discussed are relevant to the national data eco-system as it pertains to clinical data and may be useful for the refinement of that policy.

## Methodology

In July 2019 a public lecture was held on the NICD campus on POPIA and data protection for the NHLS. At that forum, staff raised with the lack of guidance for data practices and management within the NHLS. Three more public forums were planned for April and May 2020 on data protection in clinical data, health research and paediatrics. These forums were advertised to both NHLS staff and external roleplayers working with the NHLS. Parallel with these public forums interviews were to take place with individuals who were purposively selected based on their experiences in the different dimensions of working with personal health information and compliance. This included staff of the NHLS, health care professionals and partner organisations working with the NHLS, experts in security and information (both within and external to the NHLS), experts in the governance of personal health information in South Africa, and experts in the ethical issues in the use of personal health information. It was intended that these interviews and the outcomes of the public forums would then inform the initial drafting of the Code.

On 5 March 2020, the NICD recorded its first case of COVID-19 and a State of Disaster as per the Disaster Management Act 2002 was declared on 15 March 2020. The attention of those within the NHLS, and particularly the NICD as the national public health institution of South Africa, was diverted to responding to the COVID-19 pandemic. A shift in methodology was necessitated and it was decided that a draft Code would be developed by Ciara Staunton (CS) and the interviews would be subsequently held to inform further revisions of the proposed NHLS Code.

The initial draft was based on informal discussions with NICD staff on the RfA Reports in July and September 2019 and at the public forum on POPIA at the NICD campus in Sandringham, Johannesburg in July 2019. The first draft was completed in May 2020. Feedback was provided by Gayle Sherman (GS) and staff at the NICD and a revision of the Code completed in June 2020. At this stage, CS and GS identified gaps in the draft Code and areas where further expertise were required.

An interview guide (in S1 File) was developed, and a purposive selection of participants was identified by CS & GS. The interviews discussed challenges respondents faced in processing personal health information in South Africa, their perspectives on POPIA, the impact it may have on the processing of personal health information, their experiences with the processing of personal health information by the NHLS, and how the governance and oversight of personal health information by the NHLS could be improved.

NHLS staff identified as potential interviewees worked in senior management, governance, data management, and security. The NHLS Code would ultimately apply to all personal data held by the NHLS irrespective of whether the individuals processing the personal data were employed by the NHLS. The NHLS works with a considerable number of individuals and organisations who collect personal data to be processed by the NHLS, share personal data with the NHLS for clinical care, treatment programmes, surveillance, and research, and also seek access to the personal data held by the NHLS. It was therefore necessary to interview this category of individuals to inform the development of the Code. To ensure that the Code was reflective of national best practice, discussions occurring on the national management of health data and best practices in the governance of and securing of health data in South Africa were necessary. Therefore a third category of individuals with expertise in these areas, but with no link to the NHLS, were identified. In total, 31 were invited to interview: 15 were NHLS staff, 8 worked with the NHLS but were not employed by the NHLS (NHLS Affiliate), and 8 were external to the NHLS. Of these, 19 accepted participation in an interview (breakdown of respondents in Table 1). In total, 4 were NHLS staff, 7 were NHLS Affiliates, and 8 were external to the NHLS. The low response rate of many NHLS staff was due to COVID related time pressures.

**Table 1:** Breakdown of respondents.

Respondents		
Legal	4	1 NHLS staff, 1 NHLS Affiliated, 2 External
Medical Doctor	3	1 NHLS Affiliated, 2 External
Government official	3	1 NHLS Affiliated, 1 External
Social scientist	2	2 NHLS Staff
Security expert	2	2 NHLS External
Ethicist	2	1 NHLS Staff
Medical scientists	1	1 NHLS Staff
Digital health	1	1 External
Bioinformatics	1	1 External

Interviews were conducted via zoom, recorded, subsequently transcribed verbatim, and managed using NVIVO 12. A codebook was developed between CS & Kathrina Tschigg (KT). CS and KT coded 6 interviews individually with the differences discussed. This approach was chosen to have a collaborative analysis that helps to affirm but also to deconstruct and construct individual or unique interpretations through the diversity of perspectives [14]. CS then coded the remainder of the transcripts. Themes and sub-themes were developed inductively from the data and discussed between CS and KT.

Ethics approval was obtained from the Human Research Ethics Committee (Medical), University of the Witwatersrand (M200207).

## Results

There was a general awareness amongst respondents on the need to strengthen the protection of personal health information. Respondents reflected that this is broadly due to four factors. First, it is due to POPIA. It was noted that although POPIA is not the only piece of legislation that impacts on personal health information, these issues have gained prominence with the coming into force of POPIA. For most, there was agreement on the need for POPIA generally as it should improve security about personal information, guard against data breaches, and overall compliance should improve the reputation of an institution.

*“We have really struggled with compliance…and matters have profoundly come to a head in part because of POPIA… highly sensitive named patient data is not exclusively contingent on POPIA*, *there’s a gaggle of legislation*, *regulations that demand this emanates from the National Health Act”* [Government dept, 03, NHLS Affiliated]

Second, some respondents noted that the GDPR has had some influence on the strengthening of data protection in Sout Africa. This is in part because EU funders require compliance with the GDPR as a condition of funding, but also collaborators from the EU now require compliance with the GDPR. In addition, there was the belief amongst many respondents that the Information Regulator will consider and follow many of the opinions of the European Data Protection Board (EDPB).

Third, the impact of policies of funding agencies on data (that fund both treatment programmes and research), in particular the international funders of health, was highlighted by many participants. Funding agencies generally have policies in place on data management, in particular on data access and sharing. Although it was felt that Sout Africa has sufficient data protection and robust research ethics regulations in place, it was noted that funder policies on data may fill the vacuum in countries where no national data governance and data protection frameworks are in existence. In the Sout African context, it was noted that some of the policies on data access and data sharing required by international funders of health might not be in line with more local regulations and policies, particularly open access policies. Respondents noted that some feel forced to accept the conditions of funding and “import” the policies due to the lack of funding available locally.

*“There’s a much bigger issue around governance around what exactly is development aid geared toward*?*”* [Government Dept, 03, NHLS Affiliated]

Fourth, there was an awareness of the need to strengthen the protection of personal health information due to the type of personal information being processed by the NHLS and the social dynamics around it. This awareness was particularly evident in discussions around HIV, how this personal information may be used, and the need to guard against stigma and discrimination associated with HIV.

### Tensions between strengthening data protection and the use of personal health information

Despite this perceived need for POPIA, there were concerns about its implementation in the health context. In particular, concerns were expressed about its impact on patient care, treatment programmes, and research. It was noted that for individual patient care, numerous health care professionals need to have access to a patient’s identifiable personal health information. This information is often provided over the phone, via WhatsApp, via email, and patient summaries may be stored on individual computers and laptops. Concern was expressed that such methods of accessing and sharing this information may not be compliant with POPIA. Respondents asked questions about the possible legal implications of continuing with these practices, but also the impact it will have on patient care if they cannot be used going forward.

*“One of the things that I do find is the whole issue of WhatsApp*, *especially diagnostic in a clinical setting*. *For example*, *I would get a WhatsApp from a registrar about can you check on this patient with the patient name and the hospital number or the episode number*. *And so obviously that is sharing of information using a social program…It’s not sort of clear-cut in POPIA*.*”* [Medical Scientist, 01, NHLS Staff]

In the context of treatment programmes, it was noted that their success is reliant on the sharing of identifiable personal health information amongst many different actors and programme partners. This includes Department of Health (DoH) staff, who frequently rotate between different facilities, managers who monitor health programs at district or provincial level, and donor-funded healthcare workers contracted to support and mentor DoH staff. In addition to ensuring the success of these treatment programs, two respondents noted that due to the overall public investment in health in South Africa, aggregated data should be made available as an accountability measure that the funds are being well spent.

There were two main concerns about data protection in the context of treatment programmes. First, respondents involved in the administration of externally funded programmes noted that it can be impossible to meet the targets set by the treatment programs in a manner that is compliant with data protection standards. Second, concerns were expressed about the continued ability to share personal health information amongst the different organisations involved in the roll-out of these programs when POPIA comes into force. One respondent gave the example of a treatment programme that was shut down for a period of time until concerns regarding the sharing of identifiable personal health information with other third parties were addressed.

*“Maybe six*, *eight months ago*, *there was a sort of storm that came up around third party… getting access to Department of Health data to be able to track clients*, *and suddenly they shut it all down and said we shouldn’t be accessing that because we’re third parties*.*”* [MD, 02, NHLS Affiliated]

In the context of research, questions were raised about how POPIA would interact with open science. Respondents recognised that there is a real value in making the data available, but the issue now is how to do that in a manner that is compliant with the law and societal expectations.

*“It’s a very*, *very difficult and very sensitive question to answer because you want to promote open science*, *but you also want to have ethical behaviour*.*”* [Bioinformatics, 01, External]

A cross-cutting concern was that that compliance with POPIA would lead to increased bureaucracy and would disrupt processes that were perceived to be currently working well. There were also concerns that compliance may require new infrastructure, security standards, and staff training that would result in increased costs.

*“I’m just concerned* [about] *the additional barriers to research that add very few additional protections*, *because I think the research enterprise is already in this country quite competently managed”* [Ethicist, 02, External]

### Data management

It was clear from respondents that overall there is a need to rethink the management of personal health information in South Africa. As part of this, 6 issues were highlighted as needing to be addressed at a national and an institutional level: an understanding of the importance of personal information; data quality; access to, sharing and linking of data; security of personal health information; financial resources and equity of access; and knowledge, education and implementation of POPIA.

#### Importance of personal health information

Overall it was felt that the importance of personal health information is not understood at a national or a local level, and there is a need for education across all levels of the data eco-system. At a national level, the South Africa government needs to consider personal information as a resource that must be appropriately managed. There is a need to protect personal information and in particular the flow of personal information outside of South Africa. Access should not necessarily be restricted, rather it needs to be better managed. As part of this, there is a need to consider the risks and opportunities of the commercial use of personal health information and how best this should be managed.

*“The Department of Health doesn’t seem to know*. *It doesn’t seem to appreciate that data is an asset that it needs to be protected*, *that needs to be shared in a responsible way*.*”*[Digital Health, 01, NHLS Affiliated]

*“I think that the government should start taking their data resources seriously…I find a lot of conversations that are being had on a governmental level is about protecting resources*, *but…there’s not a thought of strategic management of data as a national resource and as a financial resource as well as a knowledge resource*.*”* [Social scientist, 02, External]

One problem in the overall management of personal health information is that it has been siloed into either research data, clinical data, or surveillance data, when in reality, the data are all intertwined. Viewing it in this way could result in a sharing of expertise.

A second problem that was consistently highlighted is that different databases contain personal health information but there are no unique identifiers to link patients across databases. Respondents felt there is a need to have a national strategy on the linking of these databases which could involve using patient linking algorithms on personal identifiers. Cost, privacy, and political will were noted as barriers in achieving this, but it was felt that these are issues that must be grappled with if the government wants to push digital innovation in medicine and health.

*“If we could share information between databases*, *we could actually*, *without even having to have a full electronic patient record*, *we could put together a lot of information on how to improve patient care because people do migrate through the system*.*”* [MD, 03, NHLS Affiliated]

#### Data quality

Problems relating to data quality were a common theme and there were three factors identified that contributed to that. First, there is a lack of awareness or understanding of the importance of personal health information. There was the perception that if those capturing the data understood the importance of personal health information, there would be a more accurate recording of personal health information and data quality would improve. Second, the sheer number of patients that come through the various public health care settings make maintaining a high standard of data quality challenging. It was noted that there is the possibility of multiple folders for one patient and that patient records can be lost. Poor data quality and record-keeping can affect the continuity of care. Third, it was felt that the manual process of capturing personal information can lead to inaccuracies and there were calls for standardisation of methods in capturing and recording personal health information across organisations, a move to electronic health records, and the introduction of a unique identifier for every person, including newborns at the time of birth.

*“But if there was a better understanding of personal information*, *the need for it*, *let alone the privacy*, *just the need for it from an operational standpoint and how all of this feeds into making the health system*, *I think the process would benefit because then everybody has the same understanding and the same method and methodology of how things are done*.*”* [Security, 02, NHLS Staff]

*“The capacity to ensure the highest quality and quality control and checking of queries and that kind of thing is quite hard to maintain because you do capture quite a lot of patients*.*”* [MD, 01, NHLS Affiliated]

However, it was also noted that data quality issues are often out of the hands of the data capturers. Details can change and the patients themselves may decide to give false or inaccurate information due to the stigma of their disease or that they are undocumented migrants in the country.

*“There’s still quite a lot of stigma here around HIV*. *So people do sometimes on purpose give false information*, *which means that we can’t track or trace them*.*”* [MD, 02, NHLS Affiliated]

*Access*, *sharing & linking of data*. As discussed, access to and sharing of personal health information is important for patient care, treatment programmes and research. Issues that arose included access to personal health information (that includes for linking purposes) needs to be better managed, and reluctance to share personal information by those who control and manage access to these data, needs to be addressed.

Respondents reported that there currently is a general lack of formalised policies on access to and sharing of personal health information. In the research context, REC approval is usually sufficient to be granted access to personal information. However this is not always the case and the process is often unclear. Outside of the research context, access to personal information is necessary for individual patient care, surveillance and treatment programmes but a formalised process is often lacking. Where a process is in place, generally a letter from the custodian of the database is required, and if it is held by a provincial hospital, a letter from the relevant official in the provincial DoH is required. However, respondents noted that access is usually decided by the individual who collected the personal information or a gatekeeper who is difficult to identify. Concern was also expressed that certain individuals hold considerable power in deciding access and they can block access to the personal health information without any justification or accountable process in place.

*“So it’s kind of like individuals making decisions rather than actually a more formalized process*. *It’s individuals that are influencing the process and because they are in a position of power*.*”* [Government Dept, 01, External]

It was felt that it would be best if there was a clear and transparent management process in place for accessing personal health information in South Africa. The extent to which there could be a national procedure in place was questioned due to the autonomy that the provinces have in matters relating to health.

At a more local level, it was felt that the overall management of personal health information would be strengthened if there were more formalised workflows established and the individuals responsible for compliance with POPIA clearly identified. It was noted that NHLS staff usually know their Safety Officer or Quality Officer, but not their Information Officer.

*“If you ask around who is your data compliance officer*, *because I mean*, *I can go around and I know I would know my safety officers*, *I would know who my Quality Control officer is*. *But my data officer*, *nobody can answer that*.*”* [Medical Scientist, 01, NHLS Staff]

As one way of strengthening this process, the establishment of data access committees (DAC) to manage access, sharing and linking requests was recommended. It was felt that DACs would shift decisions from individuals to an independent committee, thereby helping to get rid of internal bias in access decisions that is currently felt to exist. It was felt that DACs should be a multidisciplinary committee that assess several aspects including intellectual property (IP), ethics, historical conduct of the institution seeking access, and safeguards and procedures in event of a breach.

In the context of research, DACs would have a link with RECs, but respondents felt that they would have a separate function. In particular, they would be comprised of individuals with expertise in data protection, technical data skills, security, community representatives and, scientists, among others.

*I think it’s a very good development as a subcommittee of an ethics committee*. *It will definitely play an important role*. *I think it’s important that the terms of reference for the committee be very clear to avoid any duplication within an institution*. [Legal, 01,NHLS Affiliated]

The reluctance to sharing personal data was a point mentioned by many respondents. It was felt that this was due to previous experiences, that included a lack of acknowledgement of the local data collectors. Concerns were raised that those in high income countries (HICs) would be in a better position to analyse and use the data.

*“I have seen data*, *for instance*, *from our HIV program*, *being presented by large organizations at large international conferences with very limited acknowledgement of the fact that it was actually local people who worked very hard to see all those patients and to generate that data*.*”* [MD,03, NHLS Affiliated]

Respondents also noted that there is a need to ensure that the use of personal health information is in line with the expectations of patients specifically and the public generally. While there have been some discussions with the public and community on the use of biological samples, questions were raised over whether the cultural considerations in the use of personal health information have adequately been identified and discussed. There was the general feeling that to date there have been inadequate discussions with local communities in South Africa and it cannot be assumed that the same cultural and social considerations apply to personal information as biological samples. Such conversations were felt to be necessary for developing data management processes as conversations on data management to date have largely been driven by HICs. Specifically for South Africa, due to the bifurcation of the South African society in its access to private and public health care, people experience health, health care, and health data differently and they also experience rights differently. These experiences may affect their views on personal health information and its use. To address this, public engagement on the use of personal health information was called for.

*“Differential experiences and understandings of health and health data and health rights need to be taken into account in developing governance frameworks*.*”* [Government Dept, 02, NHLS Affiliated]

*Security of personal health information*. All respondents spoke of the need to ensure the security of personal health information. Generally, the focus was on securing digital personal health information, but some respondents did note the importance of securing other forms of personal health information, including patient files and samples. Organisational measures mentioned included introducing an access control system to authorised users only, use of unique passwords, access agreements, user agreement policies, privacy training, use of material transfer agreements and data transfer agreements. Technical measures included firewalls, encryption, VPN, anti-malware, and intrusion prevention systems. It was clear that prior to the sharing of personal health information, the purpose for which it will be known must be clear and the safeguards must be outlined. It was suggested that where possible, personal health information should be de-identified (i.e., anonymised) before sharing. However, there were questions on the extent to which this can be guaranteed for certain types of data and the impact that de-identified can have on the value of the data was noted.

*“We started off with hyper compliant de-identification*, *but that proved to be so stringent that we couldn’t work with the data*. *So that’s always one of the problems we have*, *which a lot of people will have*, *is that the more you de-identify*, *the less useful the data normally is*.*”* [Digital Health, 01, NHLS Affiliated]

The importance of implementing security measures based on a risk assessment was emphasised and it was felt that this should be repeated frequently in much the same way as quality assurance is carried out in laboratories. It was felt that security standards should be context-dependent based on what is reasonable in the circumstances. There were concerns about the impact infrastructural issues such as low bandwidth, power outages, and the shortage of personnel with the requisite security expertise, could have on data security. In particular, issues of cost could be a factor to take into consideration. For example, ISO 2700 was mentioned by two respondents as a standard which should be achieved, but it was questioned whether accreditation is something that many South African institutions can afford.

*“I guess we can have a lot of challenges*, *especially driving the knowledge to everyone*…*we might have to acquire in the environment the skills that we need in the environment to ensure that we are secure*.*”* [Security, 01, NHLS Staff]

It was clear that respondents considered the integrity of individuals who have access to personal health information to be a key factor in ensuring the security of personal health information. It is the individual who was viewed by some respondents as being the weak link in data security therefore, individuals need to understand the importance of privacy and data protection.

*“One thing that I found is that you can have the best I*.*T*. *security systems on the planet*. *But if you have users that don’t know just the basics of something like password hygiene*, *your systems are null and void*.” [Bioinformatics, 01, External]

*Financial resources and equity of access*. The additional costs associated with POPIA compliance were raised throughout the interviews. It was queried whether all institutions will be able to afford to meet these new standards. This raised concerns about whether this will favour larger and well-funded institutions with concerns that it will perpetuate inequity in accessing data.

*“If POPIA is in any way similar to GDPR…then what will happen is that all the highly resourced universities that have now been investing time in GDPR will be ready to go*. *And all…partners who may not have had the time to invest in all the systems will have to catch up and research will struggle*. *So then one is exacerbating the power inequalities of who can access the data*.*”* [Social Scientist, 01, NHLS Affiliated]

#### Knowledge, education and implementation of POPIA

A key factor in strengthening data management is to address the perceived low levels of knowledge and understanding of privacy, security, and the importance of personal health information. This includes at a national level where awareness on the importance of data management must be addressed.

The uncertainty in the application of many of POPIA’s principles to the processing of personal health information was noted by many respondents. Questions were asked as to whether decisions on restricting access to or linking of datasets were justified or due to a lack of understanding of POPIA and a fear of not being compliant with the legislation.

*“I think that people are confused and people who are confused and frightened don’t know how to take the next step forward*. *They don’t know how to implement things*.*”* [Bioinformatics, 01, External]

It was felt that the Office of the Information Regulator had the potential to have strong authority to regulate these matters. However, concerns were expressed about (the then) under-resourcing of this office and the impact that would have on the ability of the office to fulfil its mandate. There was widespread feeling amongst respondents on the need for a Code of Conduct for the use of personal health information in all contexts discussed in these interviews (i.e. clinical care, surveillance and research). It was felt that a Code could provide a useful roadmap in the use of personal health information, with clear lines of accountability and it was recommended that it should provide examples and scenarios to demonstrate how it may apply in practice. There was a concern that without such guidance, these matters will end up in court. Much of the discussion on codes focused on the need for a Code of Conduct in the research context. It was felt that there should be one Code for research as otherwise there was the concern that institutions would work in silos and each produce their own Code. This would be problematic for a number of reasons, including that more than one conflicting Code could apply to individuals who have dual appointments.

*“I think we just do need that guidance in terms of health research*. *I think something is lacking and that researchers and institutions*, *et cetera*, *will appreciate that type of clarity*.*”* [Legal, 04, External]

For those that discussed exemptions and codes, there was a preference for the use of a Code as opposed to seeking exemptions for the sector or individuals. This preference was based on the fact that the Code will be informed by the relevant industry with approval by the Information Regulator.

## Discussion

Discussions to date on POPIA have very much focused on consent and in particular the legal status of broad consent i.e. consent to data to be used for unspecified future research [7, 15]. This is perhaps reflective of the ongoing ethical debate on broad consent in research [16–20], but there has been very limited consideration of the wider issues in the use of personal health information in South Africa, particularly in the clinical context. Although broad consent, and in particular the need for clarification on the legal status of broad consent under POPIA was discussed by respondents, it was not a focus. Our findings reveal that there are issues in the management of personal health information at all levels of the national data ecosystem.

It was clear from respondents that there needs to be a more integrated approach to the management of personal health information at a national level. The potential of an integrated approach to the management of big data in health that use new and existing data sets and link them together is considerable [21], but it is often hampered by legal, technical, and political barriers [22, 23]. To achieve this, there has been a push towards making data Findable, Accessible, Interoperable, and Reusable (FAIR) [24], but this is still rare [25] and integration of data sets is an ongoing challenge [26, 27]. In South Africa, the autonomy of the provincial departments of health further challenges the possibility of a national integrated approach to data management. Despite this, there is a need for a national conversation to drive a national agenda for the governance of health information. There are a number of steps that can be taken at a national level that could address many of the issues faced at an institutional level.

First, science, technology, and innovation has been identified as key drivers of economic growth in the South Africa National Development Plan with open science and the access to and sharing of data seen as important elements of the Fourth Industrial Revolution. South Africa. Department of Science and Technology White Paper: Science, Technology and Innovation. 2019. This has yet to be translated from policy into practice and it is currently being hampered by a lack of clear and transparent processes to the management of personal health information across all sectors in South Africa. A key problem identified here is that there is a lack of awareness of the value of personal health information at a national level. This may change if the draft National Data and Cloud Policy comes into force. This draft policy recognises the importance of data and the digital economy across all sectors of society and seeks to develop “citizen centic frameworks” that will harness the potential of data and cloud computing.

It is critical that personal health information is a resource that is to be protected, but equally as a resource to be appropriately used, shared, and linked with other data sets where necessary for patient care and arguably, the wider public interest. The draft National Data and Cloud Policy perhaps addresses this somewhat as it states that *all* data generated in South Africa is the property of South Africa, but open data sharing is also a key feature of this draft policy and it also seeks to extend the application of POPIA to data and international data transfers that currently is not under its remit. How this balance between data sovereignty, data sharing and data protection will be achieved remains to be seen. Historical exploitative research, inequitable collaborations and historical use of biological samples and data has resulted in resistance or at least, hesitancy, amongst many in South Africa to the sharing of personal health information [8, 9, 28]. Discussions in these interviews indicate that there is also a reluctance to share with partners within South Africa even for programmatic purposes. This brings an additional dimension to the widely acknowledged tension between open science and data sharing that must be considered in the South African context. However, a conversation on how best to safeguard the privacy of the data subject, safeguard personal health information as a resource, while enabling the use of the personal health information in a manner that is in the best interests of South Africa needs to be held before the draft National Data and Cloud Policy comes into force. A key first step is understanding the importance and value of this personal health information to the health of South Africans.

Second, the challenges relating to the governance of personal health information are inter-departmental and cross-sectoral. There are many different government departments that have a vested interest and an important role in the management of personal health information. This includes the Department of Health, the Department of Science and Innovation, Departments of Home Affairs, and the Department of Justice. There is no one department that can take ownership of and drive this process and arguably nor should one department lead this development.

Data stewardship is an approach to the management of data that seeks to enable its use while also protecting the data and the data subject [5, 29]. The British Academy and the Royal Society’s Report on *Data Management and use*: *Governance in the 21st century* recommended the establishment of an independent National Data Stewardship Committee. Such a body would be tasked with stewarding the national data governance landscape, conducting stakeholder and public engagement, and the setting of standards and best practices. Considering the challenges relating to the governance of personal health information are inter-departmental and cross-sectoral, the establishment of an Independent National Data Stewardship Committee in South Africa may be appropriate at this juncture.

Third, respondents made it clear that there is a need for guidance on the governance and management of personal health information. It was clear from respondents that programmatic success is contingent on continued funding and trust in the system is built upon clear, transparent, and accountable policies. Governance frameworks that enables the legal and ethical use of data in a manner that prevents harm to the data subject and promotes public trust has been called for in South Africa [15]. Similarly, the importance of governance frameworks in promoting integrity, solidarity, and accountability in data sharing have been echoed elsewhere [30]. Sectoral specific codes of conduct as a means to navigate the tensions between meeting data protection standards while also enabling the sharing, accessing and linking of data have been suggested [31]. Respondents here were very welcoming of a Code of Conduct for the NHLS as an important part of enabling compliance with POPIA. This Code would, however, be applicable to data held by the NHLS only. Since the completion of these interviews, the ASSAf is developing a POPIA Code of Conduct for Research. However, these interviews make it clear that considerable issues in the use of personal health information arise outside of the research context. Considering the confusion and lack of understanding on the impact of POPIA on personal health information, the development of a more national Code of Conduct on the use of personal health information must be considered and this is an initiative that could come under a National Data Stewardship Committee. The advantage of one Code of Conduct for the processing of personal health information is that it avoids multiple and likely conflicting codes in the health sector that leads to an even more fragmented approach. It is an initiative that must also consider the realities of meeting these new data protection principles in the diverse and challenging health care settings in South Africa.

Fourth, the establishment of DACs was seen as an important and necessary step in ensuring transparent data access. DACs were seen as separate from RECs and it is important that the responsibilities of data access does not come under the functions of RECs. The primary function of RECs is to protect research participants [32, 33] whereas DACs promote data sharing in a manner that seeks to protect the data subject, their community, and the producers of data and their institution [34]. Additionally, data access requests are often made outside of the research context and a formalised process is needed for these requests. Indeed the NHLS has a formalised process in place for data requests in the research context, but there is no clear process outside of this context should personal identifiers be required.

Fifth, the economic impact of these new requirements for data protection and data management must be considered. Conversations need to be had on how to appropriately fund data management across all sectors that use personal health information and takes into consideration the realities of the different health care settings. There is the danger that less well-resourced institutions may be denied access to personal health information due to their inability to finance certain security safeguards. This risks impacting care, but also raises issues of equity and justice in access to personal health information.

Sixth, this national conversation also needs to consider how South African personal health information is managed in the international context. There are benefits to linking data sets outside of South Africa as this could provide unique insights into the origins of diseases, as well as facilitate treatment evaluation and drug development [30, 35]. Health is not just a national concern, but in a globalised and interconnected world, health risks, disease and viruses easily cross national borders [36]. A key feature of international collaborative research that many researchers in South Africa are a part of is the international sharing of data and funders and journals often do require data sharing [37–39] Thus this need for the international sharing of personal health information must be understood and a part of this national conversation. However, this must be done in a transparent manner that respects South Africa’s ethical-legal framework. In Europe, questions have been asked about the extent to which data sharing policies comply with the GDPR [37]. Here, respondents expressed some concern over the influence (and arguably pressure) of data policies that are attached to the funding of public health and health research. The power of non-governmental organisations (NGOs), philanthropic organisations and other countries to influence policy change through regulations or policies attached to conditions of funding should not be ignored [40]. The word of caution expressed by respondents who noted the power of funders in the realm of data protection must be heeded.

It is clear that POPIA places strict rules on data sharing, particularly outside of Sout Africa. International funders, NGOs and international collaborators must be aware of these new rules and respect and uphold the new legal framework on the protection of personal information in Sout Africa. Sout Africa however is one of many African countries that has strengthened its data protection framework, therefore similar concerns are likely to be felt in other African countries that are reliant on international funders to support clinical care, treatment programmes, and research. Data sharing policies and data access policies can not longer be dictated by such international bodies based on what is in their interests. Any data sharing and data access policies as a condition of funding must respect and uphold national legislation on the use of data.

Finally, it is important to note that national frameworks will not offset the need for more localised processes and workflows. It must be clarified which actors are responsible for ensuring data security and the parties concerned must be sufficiently supported for this task. While respondents often focused on the need for accountable processes and transparency in decision making and the responsibilities of others, they often failed to consider their own individual responsibility despite individual staff members being seen as the weak link. This again points to the importance of institutional workflows with a precise delegation of responsibilities and accountabilities, and also the need for education across the board on data protection, privacy, and security.

## Limitations

The findings reported here are from those who work in senior management and in the management, collection, and use of data. As per Staunton *et al* it is important that there is also community-level engagement and that the views of the data subjects themselves are sought [12]. Their perspectives on the use of their personal health information that may have been obtained through routine clinical testing, research, surveillance, provision of treatment programmes as well as other sources that increasingly includes digital tools must be considered. As part of this work, there should be an exploration of whether the cultural concerns and considerations in the use of biological samples equally apply to the use of personal health information.

## Conclusion

The implementation of POPIA to the processing of personal health information is challenging, complex, and likely costly. It is also bringing long-standing issues on data access, data linking, and data management to the fore in South Africa. The coming into force of POPIA affords us with the opportunity to address these issues and the strengthening of data protection in the context of personal health information should be seen as just one strand in the overall improvement of data management that is necessary. POPIA is about strengthening the protection of personal information, but equally its Preamble states that it is about protecting important interests that includes the free flow of information across South Africa. Personal health information must be appropriately managed to ensure the privacy of the data subject, but equally as a resource to be used in the public interest.

## Supporting information

S1 Table Breakdown of respondents. (DOCX)

S1 File (DOCX)
