# Supplementary material for: Data protection, data management, and data sharing: Stakeholder perspectives on the protection of personal health information in South Africa
Source: PLoS One. 2021 Dec 20;16(12):e0260341. doi: 10.1371/journal.pone.0260341 (PMC8687565; doi:10.1371/journal.pone.0260341)
Supplement: S1 Table — (DOCX) [file pone.0260341.s001.docx]

| **Respondents** |  |
| --- | --- |
| Legal | 4 |
| Medical Doctor | 3 |
| Government official | 3 |
| Social scientist | 2 |
| Security expert | 2 |
| Ethicist | 2 |
| Medical scientists | 1 |
| Digital health | 1 |
| Bioinformatics | 1 |

Table 1: Breakdown of respondents
