# Supplementary material for: Data protection, data management, and data sharing: Stakeholder perspectives on the protection of personal health information in South Africa
Source: PLoS One. 2021 Dec 20;16(12):e0260341. doi: 10.1371/journal.pone.0260341 (PMC8687565; doi:10.1371/journal.pone.0260341)
Supplement: S1 File — (DOCX) [file pone.0260341.s002.docx]

**Interview guide**

Thank them. Consent. May want to use examples that they provide.

1. Please tell me a bit about your current job including your duties and responsibilities

*Probe*: Do you collect, use, store, share data? What type of data? Where (i.e. physical, online)? Security standards? For what purposes? Internationally?

1. Current challenges in the collection/use/sharing of data?

*Probe:* Who collects the data for you? What are the standard of that data captor? What procedures do you follow? Rules? National guidance? International guidance? What helps guides you/institution? What are the challenges you face in the sharing of data? Who is there to provide guidance/assistance to you in the use of personal information?

1. Who should have access to the data you gather?

*Probe*: Individuals; state; researchers. For what purpose? Who should decide on access? Have you declined access request – why? Have you been declined an access request – why? Tell me about the secondary use of data: is it for the same purpose or different?

1. What other type of data do you need to do your job/work?

*Probe*: How much data do you require? Do you collect more than you need? Institutional barriers?

1. Tell me what you know about POPIA?

*Probe:* Is it necessary? Thoughts on current regulation? Do you feel that you adequately understand it? How do you think it will impact your work? Is guidance needed – broad principle based v SOP? International standards – what are the influences, what are you required to follow, policies, etc? How does it fit in with international best practice?

1. What do you think will be the challenges you face in complying with POPIA?

*Probe*: Cost of compliance. Facility have the technical capability? Any exemptions from the strict processing rules? How will it relate to other international standards? Do you think that data subjects are aware of their rights? Do you link datasets? What type; do they have unique identifiers?

1. Explain to me your role (if any) with the NHLS

*Probe:* What type of data do you use? How do you collect it, share it? Who makes the decisions and on what grounds? What are the good things about this process? What are the bad things about this process?

1. What are your thoughts on how the personal data is managed by the NHLS?

*Probe:* Is it secure? Do you have enough guidance on how to appropriately manage the data? Do you anonymise it?

1. What improvements could be made in the management of the personal data under by the NHLS?

*Probe*: Current oversight; technical improvements; security improvements; ethical compliance; legal compliance; any policies, SOPs, other guidelines that are needed.

1. Tell me about the technical, organisational, security safeguards that the NHLS should introduce.

*Probe*: What industry security or technical safeguards should be followed?

**Additional questions according to profession**

**Department of Health & NHLS staff**

1. How does this fit in with Open Science? *Probe*: How can a balance be struck?
2. What is the role for the different parties in the governance of health data? *Probe*: government, Information Regulator, institutions, researchers?
3. How well is privacy currently protected? *Probe*: in legislation, in practice, by outsiders
4. Practical challenges likely to face in complying with POPIA. Probe: costs, personnel, expertise, etc.
5. Experiences with DoH and other partners (private and public) in sharing personal information. Probe: Barriers? Processes to be followed? Type of personal that you may need/link with other data bases?

**Legal experts**

1. How will POPIA fit in with the broad regulation on health data in SA?
2. Tell me your thoughts on the current framework for the sharing of health data in SA

*Probe*: Does the current MTA deal with health data? How will POPIA change this?

1. Concerns around compliance particularly with the experiences of PAIA.

**Clinicians**

1. Particular challenges in the use of health data of children
2. Practical challenges clinics will face in complying with POPIA.

*Probe:* Describe the process of consent, gathering information, etc. How is the process different for research? Access to data – who decides?

1. Use of clinical data for other purposes such as research – what process is followed? Who decides on the use
2. Do patients have concerns about protection of personal information?

*Probe*: What discussions do you have with patients on this?

1. Experiences with DoH and other partners (private and public) in sharing personal information

*Probe*: Barriers? Processes to be followed? Type of personal that you may need/link with other data bases?
